# Analysis and prediction of human acetylation using a cascade classifier based on support vector machine

**DOI:** 10.1186/s12859-019-2938-7

**Published:** 2019-06-17

**Authors:** Qiao Ning, Miao Yu, Jinchao Ji, Zhiqiang Ma, Xiaowei Zhao

**Affiliations:** School of Information Science and Technology, Northeast Normal University, Changchun, 130117 China

**Keywords:** Lysine, Acetylation sites, Human, Support vector machine, Cascade classifier, Sequence features, Structural feature, Systematic and comprehensive analysis

## Abstract

**Background:**

Acetylation on lysine is a widespread post-translational modification which is reversible and plays a crucial role in some biological activities. To better understand the mechanism, it is necessary to identify acetylation sites in proteins accurately. Computational methods are popular because they are more convenient and faster than experimental methods. In this study, we proposed a new computational method to predict acetylation sites in human by combining sequence features and structural features including physicochemical property (PCP), position specific score matrix (PSSM), auto covariation (AC), residue composition (RC), secondary structure (SS) and accessible surface area (ASA), which can well characterize the information of acetylated lysine sites. Besides, a two-step feature selection was applied, which combined mRMR and IFS. It finally trained a cascade classifier based on SVM, which successfully solved the imbalance between positive samples and negative samples and covered all negative sample information.

**Results:**

The performance of this method is measured with a specificity of 72.19% and a sensibility of 76.71% on independent dataset which shows that a cascade SVM classifier outperforms single SVM classifier.

**Conclusions:**

In addition to the analysis of experimental results, we also made a systematic and comprehensive analysis of the acetylation data.

## Key points

1. Specifically predict acetylated lysine sites in human.

2. Combine sequence features and structural features to translate proteins into numerical vector.

3. Build a cascade classifier based on support vector machine.

4. Solve the imbalance between positive samples and negatives, and cover all negative sample information.

## Background

Protein acetylation is the process of adding acetyl groups (CH_3_CO-) to lysine residues on protein chain. As a widespread type of protein post-translational modifications (PTMs), acetylation on lysine plays a significant role in various organisms. In eukaryotes, the function of acetylation is mainly focused on the influence of cell chromosome structure and the activation of nuclear transcription factors. However, the recent study of the flux of proteins and the metabolic pathway of different species revealed that a large number of non-nuclear proteins were acetylated in the metabolic pathway which would provide an important basis for the use of various drugs or vitamins in real life. In prokaryotes, protein acetylation is mainly manifested in the following aspects: directly effecting the enzyme activity, affecting the interaction between proteins, influencing the metabolic flow.

Though acetylation is very common in biological process, knowledge of lysine acetylation is still quite limited. Since it is extremely important to understand the molecular mechanism of acetylation in biological systems by identifying acetylated substrate proteins along with acetylation sites, more and more focus is put on this field. Compared with the labor-intensive and time-consuming traditional experimental methods, such as liquid chromatography-mass spectrometry, high performance liquid chromatography assays and spectrophotometric assays [1, 2], computational approaches of acetylation sites are much more popular because of their convenience and fast speed. Recent years, many computational classifiers have been built to identify PTM sites through various types of two-class machine learning algorithms. In 2014, Lu et al. used MDDlogo to cluster positive samples and built a series of classifiers using several kinds of sequence features [3]. Deng et al. proposed a classifier called GPS-PAIL to predict HAT-specific acetylation sites for up to seven HATs, including CREBBP, EP300, HAT1, KAT2A, KAT2B, KAT5 and KAT8 [4]. There are at least a dozen of additional computational programs developed in earlier studies for the prediction of lysine acetylation sites, such as AceK, ASEB, BPBPHKA, EnsemblePail, iPTM-mLys, KAcePred, KA-predictor, LAceP, LysAcet, N-Ace, PLMLA, PSKAcePred and SSPKA [5–17].

**Fig. 1:**
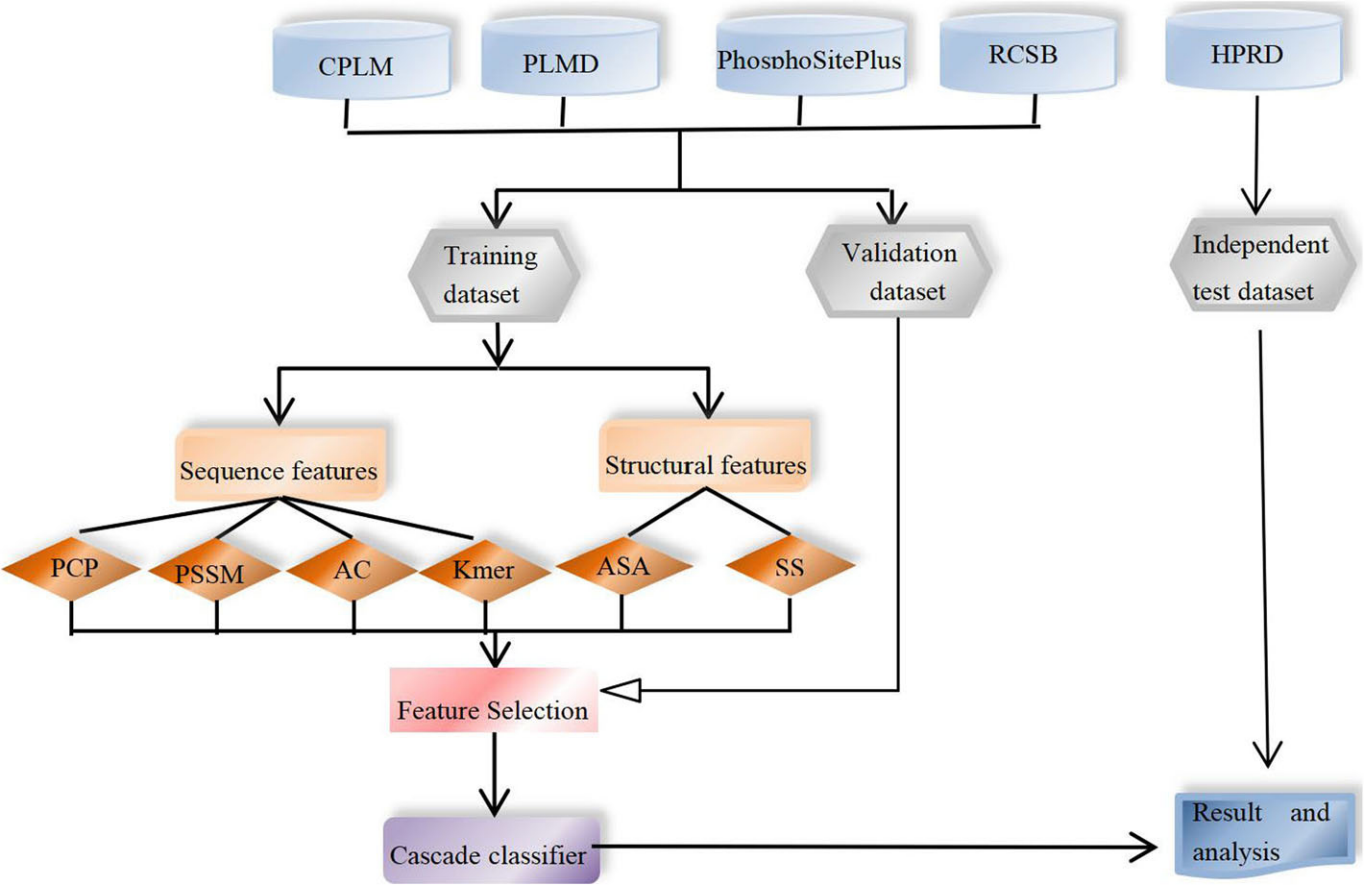
The flow chart of this method

However, these classifiers didn’t give a good solution of the imbalance between positive and negative samples. Besides, post-translational modification of proteins is species-specific, which means that different methods should be considered for the prediction of PTM sites in different organisms. Therefore, in this study, we developed a method specific to human using a cascade classifier of support vector machine to solve the imbalance problem of positive and negative samples combined with both sequence and structural feature descriptors. Finally, we made a systematic and comprehensive analysis of human acetylation data and the prediction results. The flow chart of our method is shown in Fig. 1.

## Methods

### Dataset

In this study, acetylated protein data were derived from CPLM [18], PLMD [19], PhosphoSitePlus [20], UniprotKB/Swiss-prot [21] and RCSB database [22] according to following five steps.

Step 1. First of all, we downloaded all the human acetylated protein sequences from CPLM, PLMD, PhosphoSitePlus and UniprotKB/Swiss-prot (10,146 proteins).

Step 2. Secondly, we removed proteins using CD-HIT with identity of 40%. 6834 protein sequences were left and labeled as D1.

Step 3. Next, all PDB sequences were downloaded from RCSB database and were labeled as D2.

Step 4. Then, PSI-BLAST was applied to calculate the similarity between D1 and D2. And each protein sequence in D1 only retained one matching result that had the highest score. Proteins in D1 that have no matching result were excluded.

Step 5. Finally, PDB files of proteins in D1, that were validated by X-ray diffraction and resolution less than 2.0 Å, were download from RCSB database.

After these five steps, we obtained 1213 proteins which have 3D structural information, from which 243 proteins including 451 acetylation sites and 4918 non-acetylation sites were regarded as validation dataset (used for parameter optimization and feature selection), and the rest 970 proteins including 1956 acetylation sites and 18,061 non-acetylation sites were regarded as the training dataset. To evaluate the performance of our method, we downloaded acetylated data from HPRD [23] as independent test data, in which proteins that have greater than 40% identity with training data are excluded.

Subsequently, similar to the development of other PTM site predictors [24, 25], the sliding window strategy was utilized to extract samples. A window size of 19 was adopted in this paper with 9 residues located upstream and 9 residues located downstream of the lysine sites in the protein sequence and ‘X’ was used when the number of residues downstream or upstream is less than 9.

### Features

To develop an accurate tool to predict protein acetylation sites, it is necessary and important to translate proteins into numerical vector with comprehensive and proper features. Diverse kinds of features represent different information of protein. In this study, we tested variety sequence features and structural features including physicochemical property (PCP), position specific score matrices (PSSM), auto covariation (AC), residue composition (RC), secondary structure (SS) and accessible surface area (ASA).

#### Physicochemical property (PCP)

AAindex is a database which includes amino acid mutation matrices and amino acid indices [26]. Removing 13 PCPs that include the value “NA”, 531 PCPs are available. An amino acid index is a set of 20 numerical values on behalf of the specificity and diversity of structure and function of amino acids. PCPs have ever been successfully used to predict many protein modifications in previous papers, such as S-glutathionylation and acetylation [27]. Character ‘X’ was represented by ‘0’ in each kind of physicochemical property. For each physicochemical property, we built a classifier based on it, and test its performance with validation data. Finally, we chose four kinds of physicochemical properties that have the best performances (compareing their Matthew’s correlation coefficient value), activation gibbs energy of unfolding, pH 7.0 [28], activation gibbs energy of unfolding, pH 9.0 [28], normalized flexibility parameters (B-values) for each residue surrounded by one rigid neighbours [29], averaged turn propensities in a transmembrane helix [30].

#### Position specific scoring matrices (PSSM)

The evolutionary conservation is one of the most important aspects in biological analysis, and residues with stronger conservation may be more important for protein function. PSI-BLAST [31] is a tool to calculate the conservation state of specific residues. In this work, we used PSI-BLAST against the swissprot protein database to calculate position specific scoring matrices (PSSM), which is a kind of feature that regarding the evolutionary conservation of a protein. PSSM has been widely used in some other prediction problems [32–35] and obtained satisfactory results. In PSSM, each residue in peptide had 20 conservative states against 20 different amino acids, so we can get 380 (=19*20) dimension features.

#### Auto covariation (AC)

There are many interactions between amino acids in proteins, and the physicochemical properties of proteins can reflect these interactions. Auto convariation variable [36, 37] represents the correlation of the same property between two residues separated by a fixed value, that we called lag, which means the distance between two sites. Here, proteins are replaced by four kind of physicochemical properties which we mentioned in chapter 2.2.1. The calculation formula of AC value is as follows.

1 $$ {X}_{i,j}=\frac{p_{i,j}-{p}_j}{S_j} $$

First, normalize physicochemical properties to zero mean and unit standard deviation (SD) according to:

in which j means different physicochemical properties, P_i,j_ is the j-th descriptor value for i-th amino acid, P_j_ is the mean of j-th descriptor over the 20 amino acids and S_j_ is the corresponding SD. Then,

2 $$ {AC}_{\lg, j}=\frac{1}{n-\lg}\sum \limits_{i=1}^{n-\lg}\left({X}_{i,j}-\frac{1}{n}\sum \limits_{i=1}^n{X}_{i,j}\right)\times \left({X}_{\left(i+\lg \right),j}-\frac{1}{n}\sum \limits_{i=1}^n{X}_{i,j}\right) $$

Where i is the position of protein sequence, j is one of the residues, n is the size of the window, lg is the value of lag. We have chosen two lag values, 1 and 2.

#### Residue composition (RC)

Residue composition [38] represents the occurrence frequencies of different amino acid pairs in one subsequence. It is a good representation of the local composition of protein sequences. In this work, the dimension of residue composition is 20. The matrix includes the frequencies of 20 amino acids (“A”, “C”, “D”, “E”, “F”, “G”, “H”, “I”, “K”, “L”, “M”, “N”, “P”, “Q”, “R”, “S”, “T”, “V”, “W”, “Y”).

#### Secondary structure (SS)

Protein secondary structure reflects the function of protein and impacts many kind of protein reactions [39]. Secondary structure includes alpha helix, beta bridge, strand, helix-3, helix-5, turn and bend. DSSP is a powerful tool to compute the secondary structure for each residue. DSSP [40] gives “H”, “B”, “E”, “G”, “I”, “T” and “S” as output which indicate alpha helix, beta bridge, strand, helix-3, helix-5, turn and bend. In this work, “0000001”, “0000010”, “0000100”, “0001000”, “0010000”, “0100000”, “1,000,000” stand for “H”, “B”, “E”, “G”, “I”, “T” and “S”, respectively, and “X” is represented by “0000000”.

#### Accessible surface area (ASA)

As a key property of amino acid sites, accessibility surface area plays a crucial part in protein function [41] because biological reaction always happens on the surface of proteins. Values of the accessible surface area (ASA) for residues from PDB were calculated using the surface_racer_5.0 with the 1.4 Å rolling probe.

### Performance assessment

Four intuitive evaluation indexes were derived from Chou’s symbols introduced for studying protein signal peptides [42], and they have been successfully used in some papers [43–49]. Thus, we utilized these four indexes to evaluate the proposed predictor: sensitivity (Sn), specificity (Sp), accuracy (Acc), Matthew’s correlation coefficient (MCC). And the four measurements are defined as following:

3 $$ Sn=\frac{TP}{TP+ FN} $$

4 $$ Sn=\frac{TP}{TP+ FP} $$

5 $$ Acc=\frac{TP+ TN}{TP+ TN+ FP+ FN} $$

6 $$ MCC=\frac{TP\times TN- FP\times FN}{\sqrt{\left( TP+ FN\right)\times \left( TN+ FP\right)\times \left( TP+ FP\right)\times \left( TN+ FN\right)}} $$

where *TP* and *TN* mean the number of truely identified acetylation sites and non-acetylation sites. *FN* is the number of the acetylation sites incorrectly predicted as non-acetylation sites, and *FP* represents the number of non-acetylation sites incorrectly predicted as acetylation sites.

### Feature selection scheme

Varied features are often redundant and some features are noisy and lead to negative impacts, so it is necessary to remove the irrelevant and redundant features from original feature set using an efficient feature selection method. In this study, we performed a two-step feature selection method to select the optimal feature subsets. After comparison among different evaluation index, we find that mRMR (maximum relevance and minimum redundancy) [50] can give the best result for feature selection. The detailed steps of feature selection method are as follows:

1. For the first step, mRMR value was calculated to estimate the relevance and redundancy between features. Then, we ranked these features based on mRMR value, and picked out the top 300 features.
2. Secondly, features in ranked list were added one by one into feature subset, and we built models on these feature subsets.
3. Then, validation dataset was used to evaluate the performance of these feature subsets.
4. In the end, the feature subset that has the best performance was the optimal feature subset.

In this study, we regarded MCC value as the evaluation performance in feature selection because MCC value is a comprehensive evaluation index for positive and negative samples.

### Cascade classifier

Support vector machine (SVM) is a widely used machine learning algorithm based on statistical learning theory [51]. For actual implementation, LIBSVM package (version 3.0) [52] with radial basis kernels (RBF) is used, where the kernel width parameter γ represents how the samples are transformed to a high dimensional space.

**Fig. 2:**
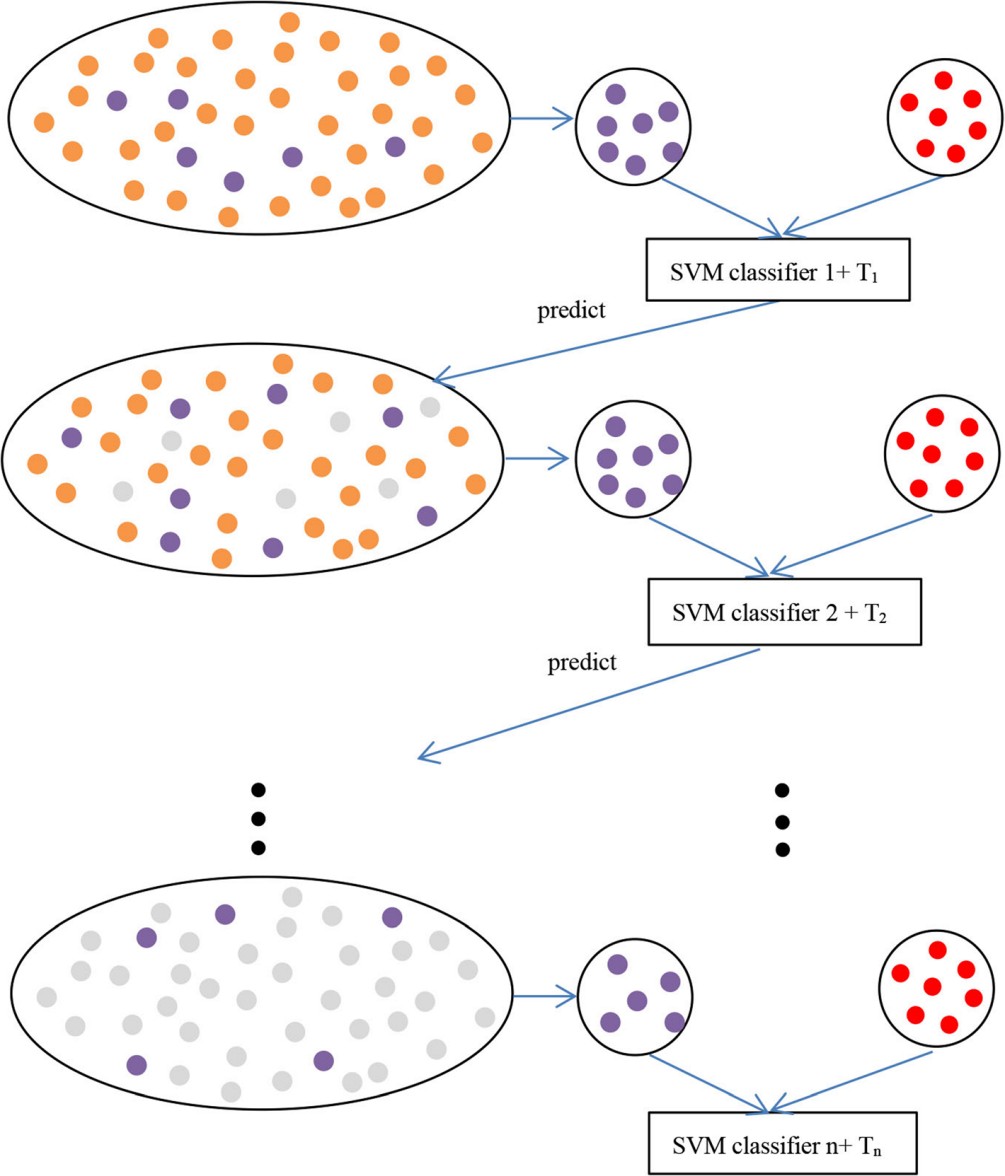
The process of cascade SVMs. Red dots are positive samples. Orange dots are non-acetylation samples. Purple dots are selected negative samples. Grey dots are non-acetylation samples that are correctly predicted and deleted

However, traditional SVM also suffer from the problem of imbalance training dataset. If all the non-acetylation sites are regarded as negative samples, the prediction results will be biased towards the negative samples and the accuracy is greatly reduced. Enlightened by the method proposed in Wei’s work [53], we built a cascade classifier based on SVM to predict acetylation sites. Figure 2 shows the process of the cascade SVMs and following is the step of building this classifier, in which PD represents positive data, TND represents total negative data and ND represents subset of negative data (the same amount of samples as PD).

Step1. Randomly select a subset of ND from TND and generate a balanced classifier S_i_ with PD and ND.

Step2. Test PD and TND with classifier.

Step3 Sort the decision value of PD from large to small and the 0.95*Mth decision value of PD is regarded as threshold T_i_ (M is the number of acetylation samples in PD).

Step4. Non-acetylation samples whose decision value is lower than T_i_ are excluded from TND, and (S_i_, T_i_) form the ith layer of cascade classifier.

Step5. Select non-avetylation sites from TND that have lower decision value as new ND, and generate a new classifier S_i + 1_ with PD and ND.

Step6. Repeat Step2–5 until less than 0.05*18061(the number of original TND) can be removed from TND.

0.95*Mth decision value of PD as threshold means that we allow 0.05 times positive samples to be predicted incorrectly in each round. In this case, if less than 0.05 times negative samples can be correctly predicted, the average value of Sp and Sn will be less than 0.5, then we should stop.

Finally, we get a cascade classifier containing n SVM classifiers, {(S_1_, T_1_), (S_2_, T_2_),..., (S_n_, T_n_)}. For a query sample q, it will be predicted from (S_1_, T_1_) to (S_n_, T_n_) orderly. If the sample q is predicted as the negative sample at any layer i, Deci_q_ < T_i_, the prediction will terminate, and q is classified as non-acetylation site, or it is transferred to i + 1 layer for further prediction. It will be classified as acetylation site only if all the SVM classifiers predict it as positive sample.

## Results

### Comparison based on features

To develop an accurate tool to predict protein acetylation sites, it is necessary and important to translate protein with comprehensive and proper features into numerical vector. Sequence features are commonly used in prediction because protein sequences are easily available. However, sometimes sequence information is not enough to describe the characteristic of proteins or amino acids, because proteins are three-dimensional, not only a chain, and the 3D structure is closer to the real conformation of proteins. Structural features are used to depict spatial information of amino acids.

In this study, we tested several features, including sequence features (PCP, PSSM, AC, RC) and structural features (SS, ASA). To verify the importance of structural features, we made a comparison between sequence features and combination features, and the performances are listed in Table 1. Combination features get a higher performance on Sn, Sp, Acc and MCC than sequence features, which indicates that structural features is significant and useful in prediction.

**Table 1:** Comparison between sequence features and combination features (sequence and structural features)

	Sn(%)	Sp(%)	Acc(%)	MCC
Sequence features (PCP + PSSM+AC + RC)	70.66	62.15	66.41	0.119
Sequence and structural features (PCP + PSSM+AC + RC + SS + ASA)	76.71	72.19	74.45	0.19

### Analysis of sequence features

**Fig. 3:**
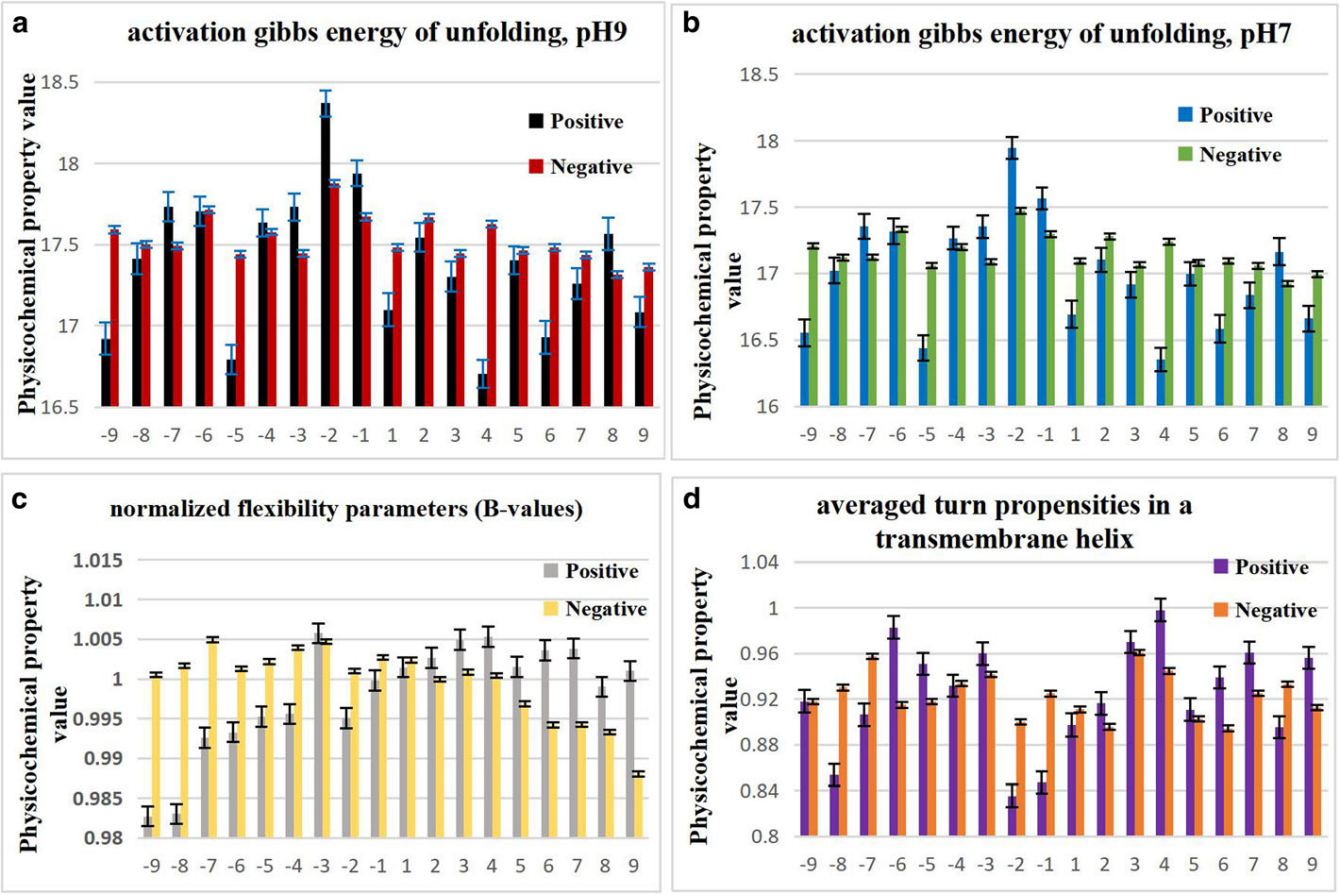
The average values of four physicochemical properties around the center residue in positive dataset and negative dataset, respectively. (**a**) is for activation gibbs energy of unfolding, pH9, (**b**) is for activation gibbs energy of unfolding, pH7, (**c**) is for normalized flexibility parameters(B-values), and (**d**) is for averaged turn propensities in a transmembrane helix

We calculate the average values and standard errors of four physicochemical properties around the center residue in positive dataset and negative dataset, respectively, and the results are shown in Fig. 3.

As shown in Fig. 3(a)(b)(c)(d), we can see that positions close to the center lysine have distinctly different values of all these four physicochemical properties. Especially in Fig. 3(a) and (b), positions in the upstream and close to lysine residues have greater values in positive dataset than in negative dataset while in the downstream, positive values are weaker. Figure 3(a) and (b) represents the activation gibbs energy of unfolding in pH 7.0 and in pH 9.0, so we can conclude from the above results that acetylation may change the direction of the unfolding process from one side to another side.

**Fig. 4:**
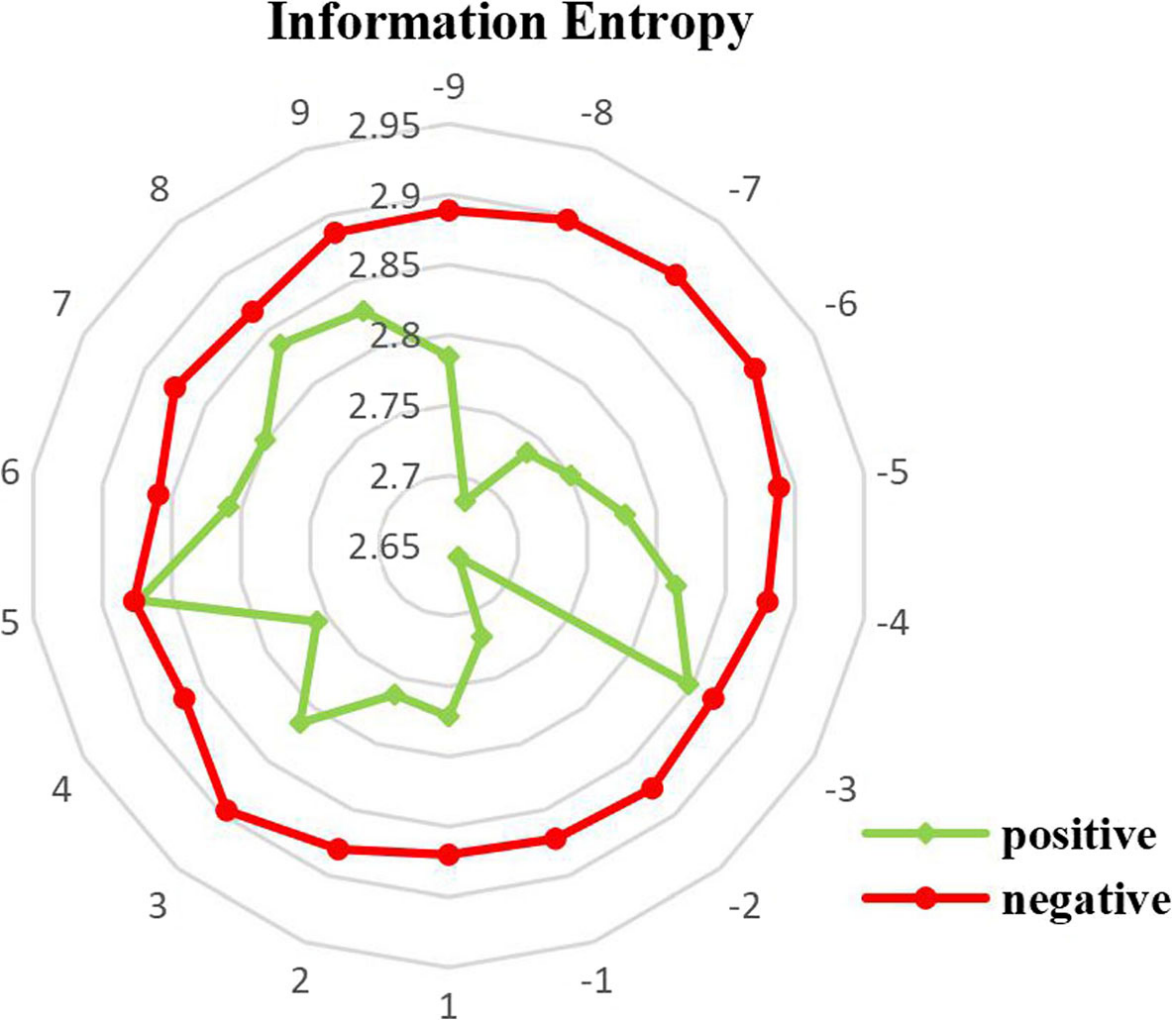
Comparison of conservation in each position between acetylated and non-acetylated peptides by information entropy values

The evolution history represents important information of a residue, and evolution information reflects the conservation information because a conserved position is more difficult to be replaced. We calculated the information entropy (IE) of positions in acetylated peptides and non-acetylated peptides, and results are shown in Fig. 4. Comparison between acetylated and non-acetylated peptides indicates that residues around acetylation sites are more conservative than those in the flanking position of non-acetylation sites, especially in the downstream.

**Fig. 5:**
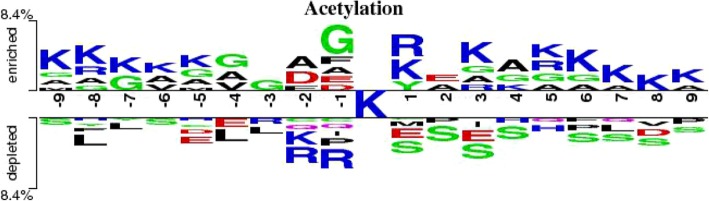
Two sample logos of the compositional biases around acetylation sites compared to non-acetylation sites. Statistically significant symbols are plotted using the size of the symbol that is proportional to the difference between the two samples. Residues are separated in two groups: (i) enriched in the positive sample, and (ii) depleted in the positive sample. Color of symbols depends on the polarity of the side chain groups in corresponding amino acids

Figure 5 shows the distribution of amino acids around center lysine. Figure 5 shows that the distribution of amino acid residues between acetylation and non-acetylation are distinct. In acetylation data, lysine (K) is enriched around acetylated lysine, especially on position 1. While in non-acetylation data, serine (S) is enriched, especially on position 1, 2, 3 and 4. Thus, it is necessary to utilize frequency-dependent feature, RC, and position-dependent feature, AC, to represent the characteristics of samples.

### Analysis of structural features

**Fig. 6:**
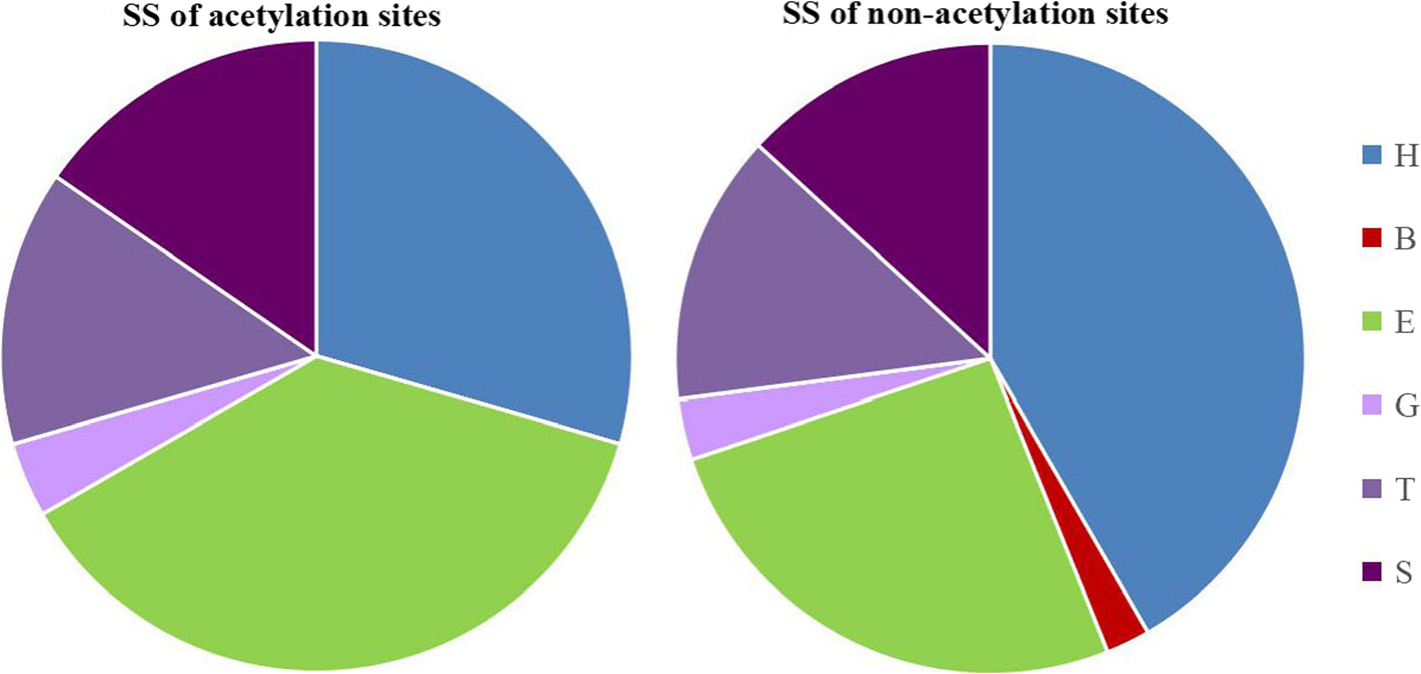
The frequency of different kinds of secondary structure in acetylation site and non-acetylation site

We evaluate the frequency of different kinds of secondary structure in acetylation site and non-acetylation site, which is defined as:

7 $$ {F}_i=\frac{N_i}{N},i=\left\{H,B,E,G,I,T,S\right\} $$

where *N*_*i*_ is the number of alpha helix, beta bridge, strand, helix-3, helix-5, turn or bend and N is the number of acetylation site or non-acetylation. The result is detailedly shown in Fig. 6.

The frequency of alpha helix on human acetylation sites is less than that on non-acetylation sites, and the frequency of strand on acetylation sites is greater than that on non-acetylation sites, which we can infer that acetylation is more likely to occur in strand region. In addition, obviously, some non-acetylation sites are in beta bridge region while no acetylation sites are beta bridge structure. Based on this phenomenon, we surmise that maybe it is extremely acetylation to happen on beta bridge region. These analyses may offer some new clues for the structural patterns surround the acetylation sites.

**Fig. 7:**
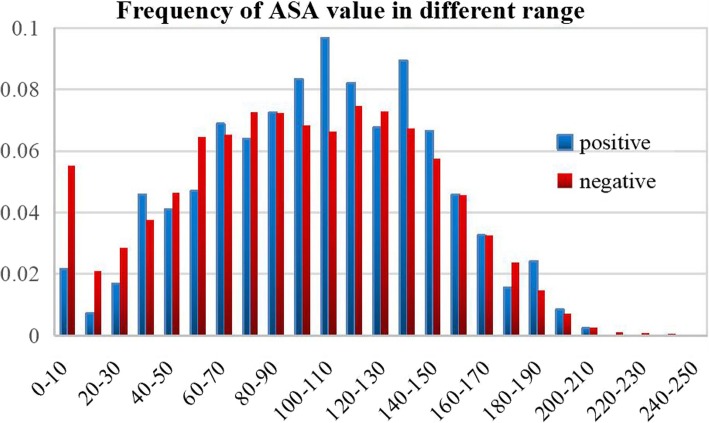
Comparison of frequency of accessible surface area between acetylation sites and non-acetylation sites

Accessible surface area represents the exposed area in protein spatial structure, and biological reaction always happens on the surface of proteins. We statistically calculate the frequency of accessible surface area value in different numerical range of acetylated peptides and non-acetylated peptides, respectively, shown in Fig. 7. As described in Fig. 7, the available surface area values of acetylation sites are concentrated between 60 and 150, and most of the frequency values of acetylation sites in this range are greater than non-acetylation sites. However, non-acetylation sites have advantage in low accessible surface area values, from 0 to 60, especially between 0 to 10. We can explain this appearance by reasonable conjecture that the larger the area exposed to the surface, the more likely the acetyl enzyme come into contact it, and if a lysine site is buried in a protein, it will have little chance to take part in the reaction. Therefore, lysine sites with greater accessible surface area are more likely to be acetylated.

### Optimal feature selection

**Fig. 8:**
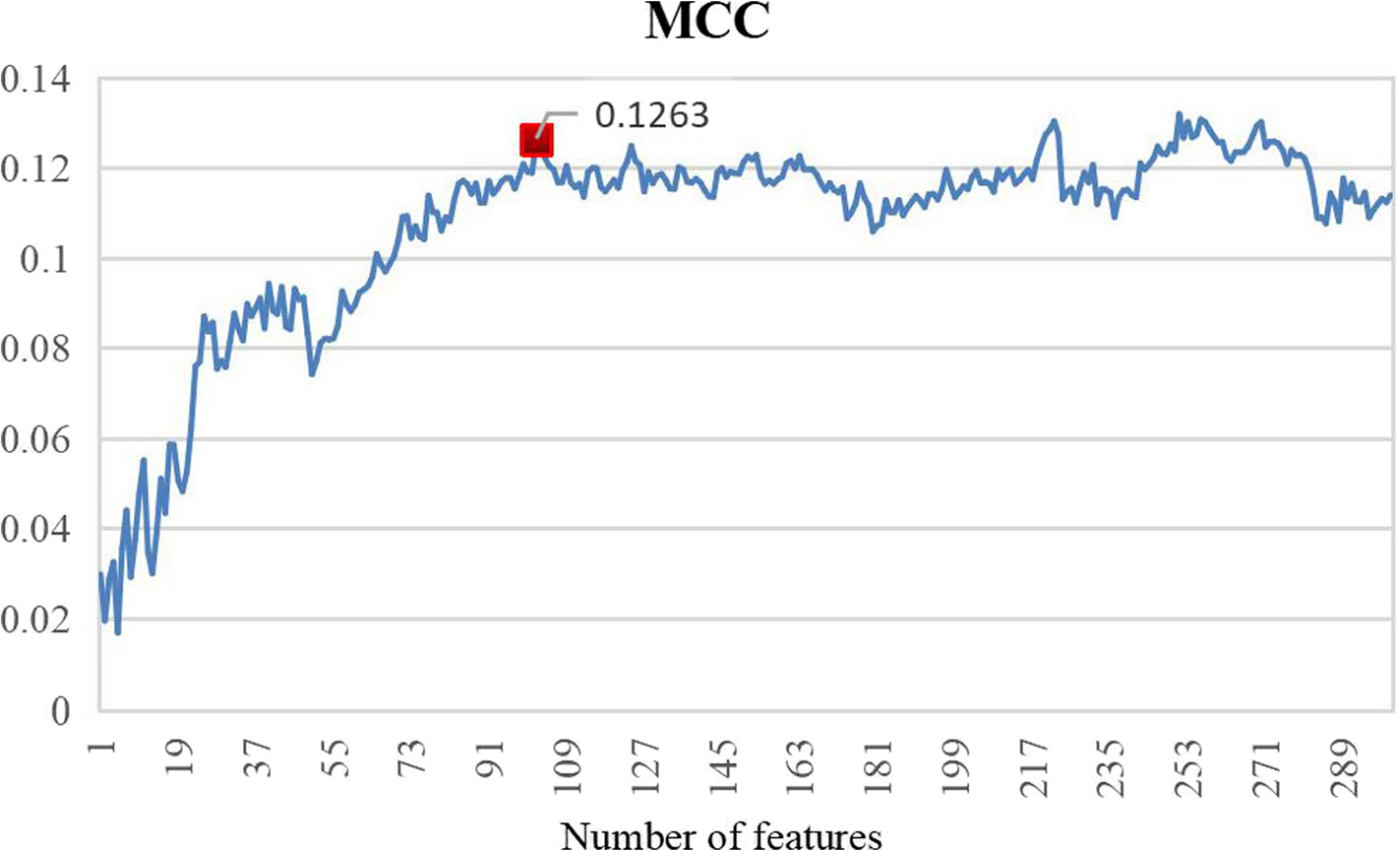
MCC curve of different number of features in final feature set

In this study, we employed a two-step feature selection scheme. In the first step, we calculate the mRMR of all features, respectively, and these features are ranked in a list according to fisher-score. Secondly, the first feature is regarded as the basic feature subset and we added features one by one into feature subset from ranked list. In the end, the optimal feature set contains 102 features and the MCC value of different number of features is shown in Fig. 8. Besides, we make a comparison of performance between before feature selection and after feature selection, shown in Table 2. Obviously, not only MCC value, also other performances are improved after feature selection. Besides, the feature dimension is greatly reduced (632 dimensions before feature selection and 102 dimensions after feature selection), which will increase the speed of prediction and save a lot of computational cost.

**Table 2:** Comparison of performance between before feature selection and after feature selection

	Sn(%)	Sp(%)	Acc(%)	MCC	Dimension
Before feature selection	63.19	52.58	57.88	0.087	632
After feature selection	69.18	53.58	61.38	0.1263	102

### Cascade classifier result

In computational methods, most of machine learning algorithms are sensitive to ratio of positive and negative samples. In this study, there are 18,061 non-acetylation sites and 1956 acetylation sites in our training dataset, nearly 10:1 for ratio of negative and positive data, so we construct a cascade classifier based on SVM to solve the imbalance problem between positive data and negative data.

To verify if cascade classifier effectively improved the prediction performances, we compare the performances of cascade classifier and single SVM classifier on independent test dataset, and the results are shown in Table 3. As listed in Table 3, single SVMs always predict a lower Sn value, Acc value and MCC value no matter trained on all training data or trained on balance training dataset. After constructing a cascade classifier based on SVMs, general performance is obviously increased. Single SVM trained on balance training dataset gets a Sn value that is not too bad, but a relatively poor Sp value, which may because negative samples used for training are only a part of all negative samples, and contains only partial information. Though Single SVM trained on all training dataset utilizes all negative samples, it results in severe sample imbalance, therefore, the Sn value is very bad. The cascade classifier not only contains almost all negative sample information, but also effectively solves the problem of sample imbalance, so it gets the best results.

**Table 3:** Performances of cascade classifier and single SVM classifier

	Sn(%)	Sp(%)	Acc(%)	MCC
Single SVM trained on all training dataset	0.91	100	50.45	–
Single SVM trained on balance training dataset	69.18	53.60	61.39	0.08
Cascade Classifier	76.71	72.19	74.45	0.19

### Comparison with exiting methods

To further evaluate the performance, we compared our method with other published acetylation prediction methods, LAceP [13], PLMLA [9], ASBE [17] and GPS-PAIL [4]. Initially, we selected 5 exiting methods to make comparison, but the web server of another method, PSKAcePred [11], can not be used. We put our independent testing dataset on other four methods and obtained the prediction results, shown in Table 4. Sn, Sp, Acc and MCC are used to measure the performance.

**Table 4:** Comparison between other method and our method based on independent testing dataset

	Sn(%)	Sp(%)	Acc(%)	MCC
ASEB	70.95	22.87	46.91	0.01
GPS-PAIL	16.41	83.12	49.77	−0.003
LAceP	66.67	43.89	55.28	0.037
PLMLA	56.76	47.38	52.07	0.015
Our method	76.71	72.19	74.45	0.19

As we can see from Table 4, LAceP get the best performance (Sn is 66.67%, Sp is 43.89%, Acc is 44.63% and MCC is 0.037) among ASBE, GPS-PAIL, LAceP and PLMLA, while our method achieve a Sp of 72.19%, a Sn of 76.71%, an Acc of 72.35% and a MCC of 0.19, which were much better than other four methods’ performance.

**Fig. 9:**
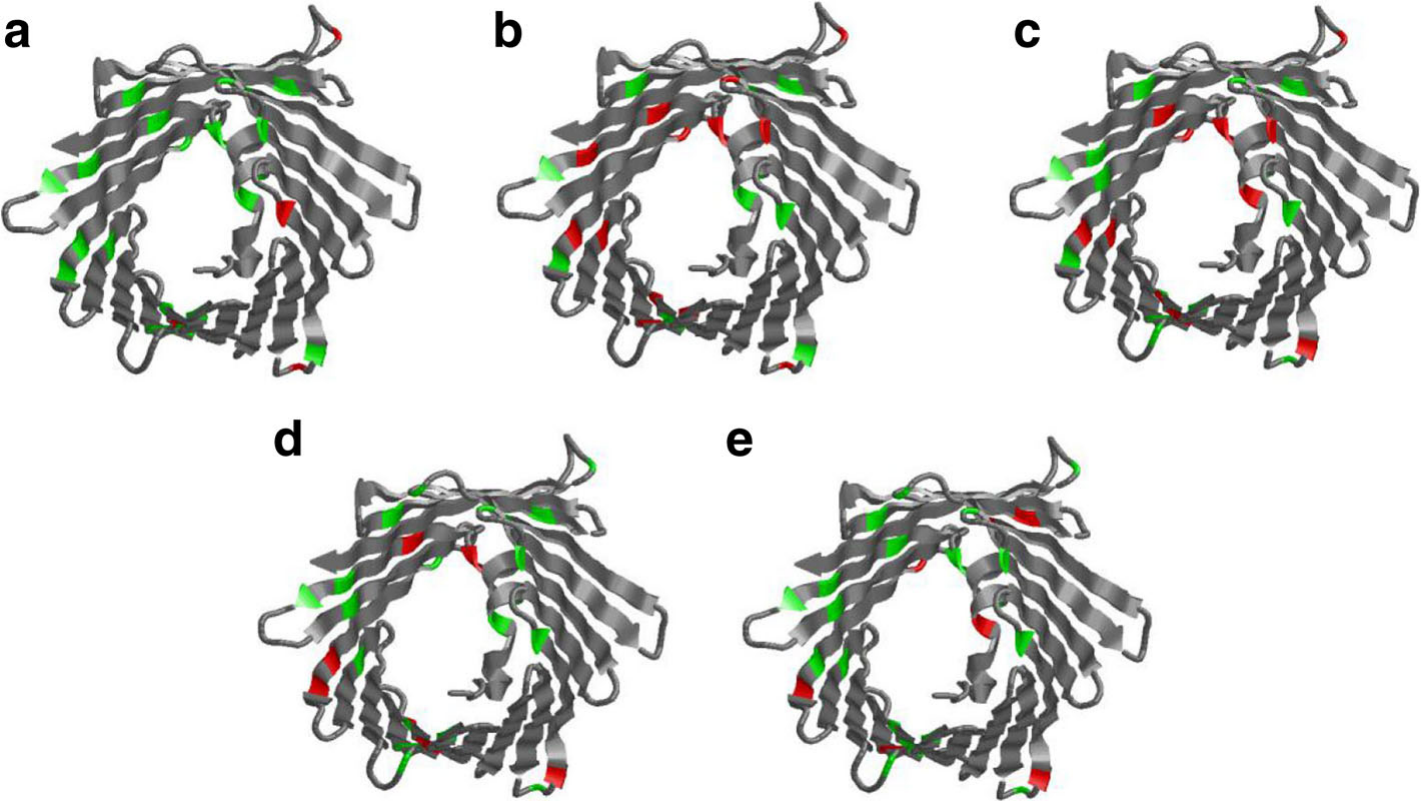
Detailed comparison between our method and LAceP based on protein P45880. **a** is the predicted result of our method and **b** is the predicted result of LAceP, **c** is the predicted result of ASBE, **d** is the predicted result of GPS-PAIL and **e** is the predicted result of PLMLA. Green parts in this figure mean correctly classified lysine sites, and red parts mean uncorrectly classified lysine sites

Then, we make a detailed comparison of the predicted results on a protein (P45880). Figure 9 describes the specific predicted results, in which green represents the correctly classified lysine sites and red represents the incorrectly classified lysine sites. We can clearly see that green sites occupy a large proportion and we can correctly classified many lysine sites that LAcep incorrectly classified. Besides, our method also has good prediction accuracy in the helix and sheet structures.

The promising performance and the conclusion from Fig. 9 demonstrate that our method was particularly useful for protein acetylation prediction than other methods.

### Comparison between different species

Due to the specificity among species, different methods should be developed for different species to predict acetylation sites. Our Method is proposed only for prediction of acetylation sites in human proteins. To verify whether our method suit for other species, we selected two mammals to test, because mammals have closer relation with human than prokaryotes or non-mammals. We obtained acetylated proteins of Mus.musculus and Rattus.norvegicus from database mentioned in 2.1, and process data in the same way as section 2.1. Then, we test these two species by our method, and the results are listed in Table 5. From Table 5, we can obviously observe that the performances of Mus.musculus and Rattus.norvegicus are not satisfactory, no matter on Sn, Sp, Acc or MCC.

**Table 5:** Comparison of performances between Homo.sapiens, Mus.musculus and Rattus.norvegicus

	Sn(%)	Sp(%)	Acc(%)	MCC
Mus.musculus	45.78	67.91	56.85	0.089
Rattus.norvegicus	57.63	56.82	57.23	0.074
Homo.sapiens	76.71	72.19	74.45	0.19

**Fig. 10:**
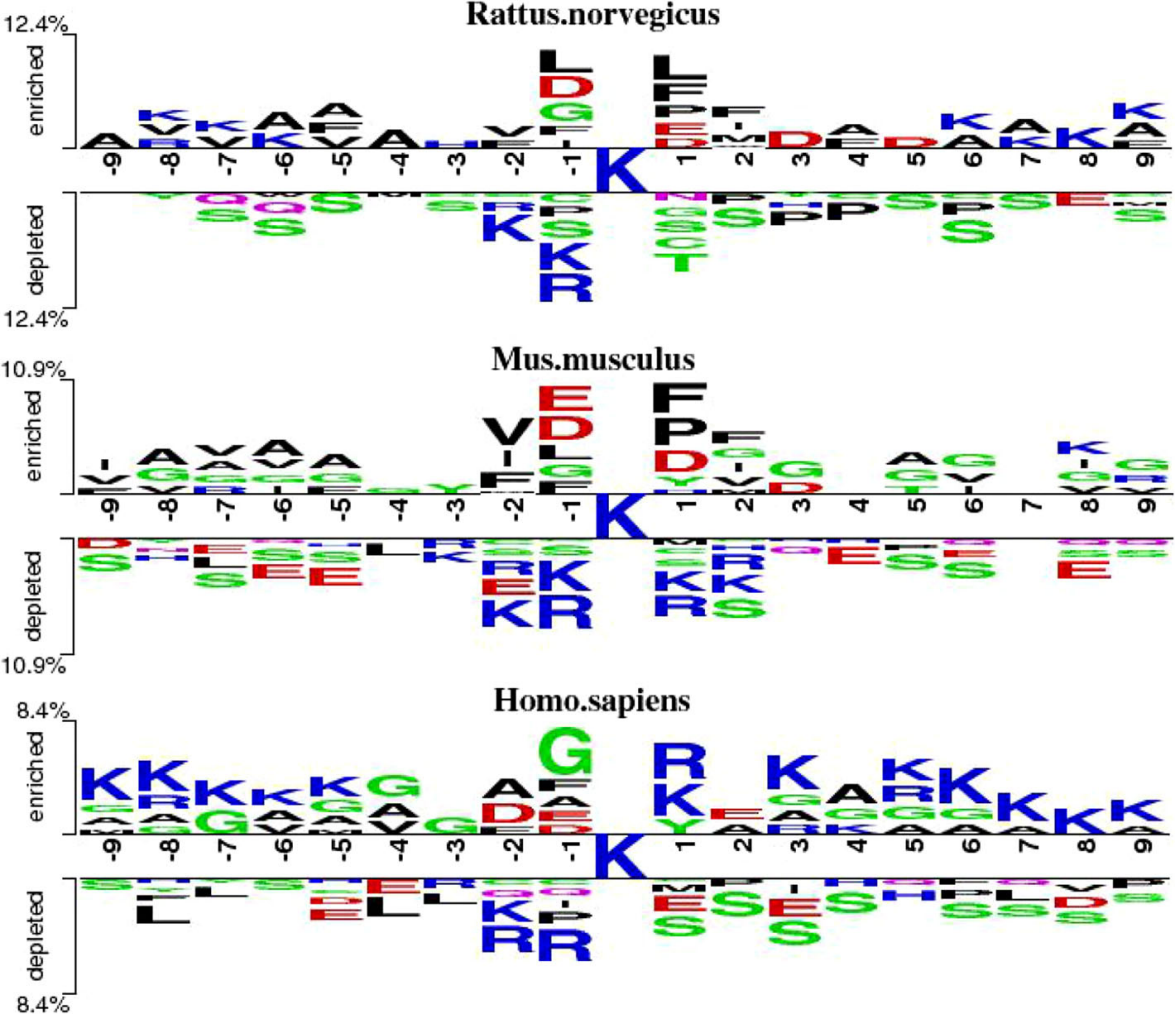
Two sample logos of the compositional biases around acetylation sites compared to non-acetylation sites in Homo.sapiens, Mus.musculus and Rattus.norvegicus

To explain it, we drew compositional biases around acetylation sites compared to non-acetylation sites in Homo.sapiens, Mus.musculus and Rattus.norvegicus, in Fig. 10. We can see that among the three species, the distribution of amino acids around center lysine is very different, especially in Homo.sapiens and the other two species, which may lead to different mechanisms of lysine acetylation. Therefore, different species may require different methods of classification.

### Gene ontology analysis of acetylated proteins

**Fig. 11:**
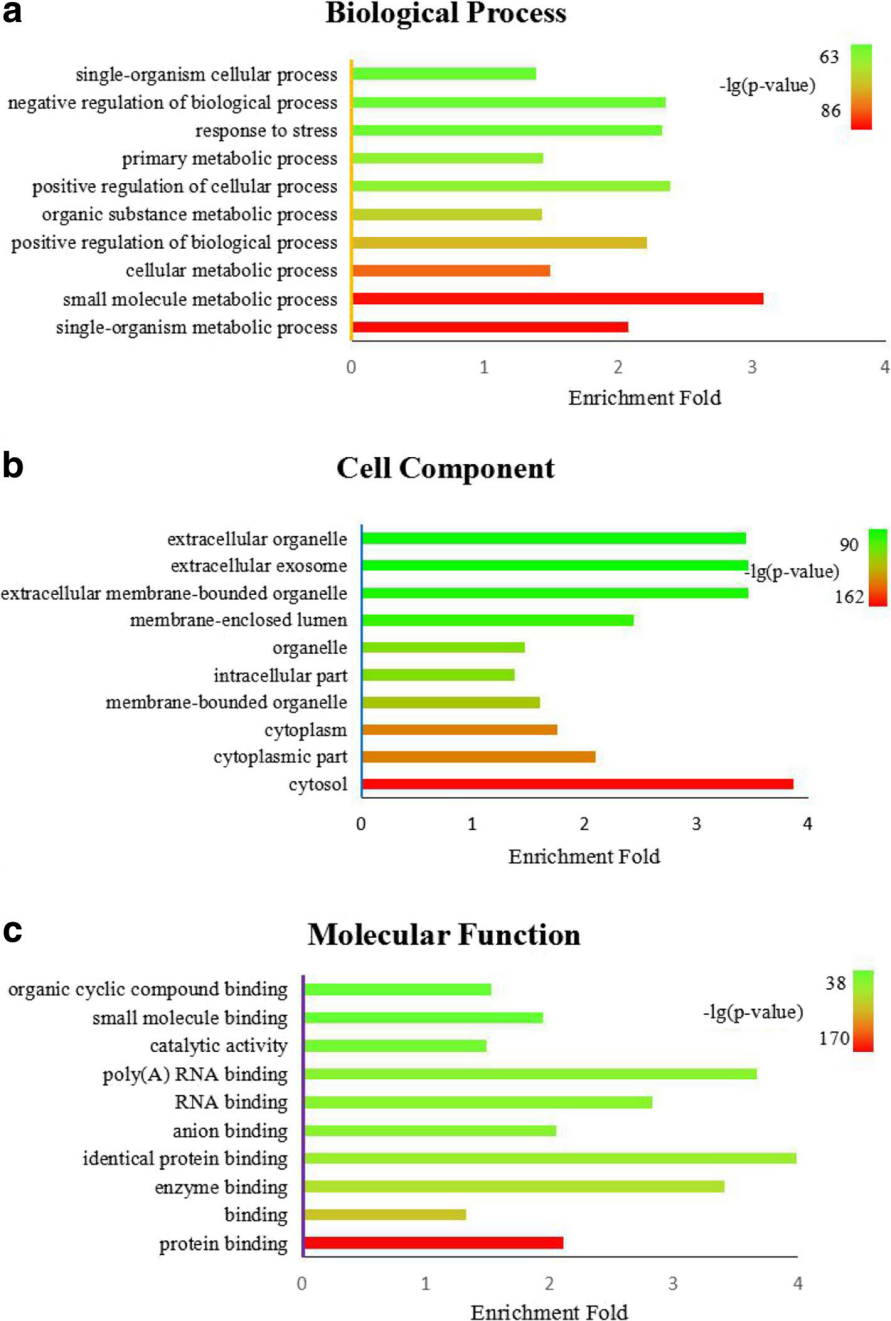
The top 10 statistically over-represented terms of biological processes, cell component and molecular functions (*p*-value< 0.01). X-axis represents enrichment fold and Y-axis means entries of biological processes, cell component and molecular functions. Different color represents different *p*-value

We statistically analyze the enriched biological processes, cell component and molecular functions with the gene ontology (GO) annotations with Fisher-exact test for acetylated proteins, of which the top 10 statistically significant terms of these three criteria are shown in Fig. 11 (*p*-value< 0.01).

We clearly find that acetylation prefers to occur at diverse metabolic process because among the top 10 biological process, 5 terms are related to metabolic process, including primary metabolic process (GO:0044238), organic substance metabolic process (GO:0071704), cellular metabolic process (GO:0044237), small molecule metabolic process(GO:0044281), single-organism metabolic process (GO:0044710). It has been reported that acetylation may play an important role in the development of cardiovascular diseases through metabolic regulation [54]. Metabolic processes that we found from GO analysis may be entry points for studies on the correlate acetylation with disease. Besides, 3 GO terms are related to regulation, including negative regulation of biological process (GO:0048519), positive regulation of cellular process (GO:0048522), positive regulation of biological process (GO:0048518). Negative regulation of biological process means any process that stops, prevents, or reduces the frequency, rate or extent of a biological process, positive regulation of cellular process any process that activates or increases the frequency, rate or extent of a cellular process, any of those that are carried out at the cellular level, positive regulation of biological process means any process that activates or increases the frequency, rate or extent of a biological process. From this observation, we can infer that acetylation is a key that can active or stop some biological processes.

As for cell component, the top three most significant GO terms are cytosol (GO:0005829), cytoplasmic part (GO:0044444) and cytoplasm (GO:0005737), and intracellular part (GO:0044424) is also in top 10, which are all positions in cellular. And among the top 10 biological process, 3 terms are different extra cellular positions including extracellular membrane-bounded organelle (GO:0065010), extracellular exosome (GO:0070062) and extracellular organelle (GO:0043230). These observations indicate that acetylated proteins are active no matter in cellular or out of cellular.

For molecular function, we can obviously find from that 9 terms among the top 10 GO terms are about binding (protein binding (GO:0005515), binding (GO:0005488), enzyme binding (GO:0019899), identical protein binding (GO:0042802), anion binding (GO:0043168), RNA binding (GO:0003723), poly(A) RNA binding (GO:0044822), small molecule binding (GO:0036094), organic cyclic compound binding (GO:0097159)). We can infer from it that acetylation may promote binding between proteins, various ligands and compounds which may cause a lot of diseases. Besides, enzyme have high specificity and catalytic efficiency to their substrates, and catalytic activity (GO:0003824) means catalysis of a biochemical reaction, both of which are essential for a lot of biological processes and ensure that the intricate biological processes within the cell can proceed in an orderly manner.

Taken together, these observations show that acetylation plays an indispensable role in human body.

### KEGG analysis of acetylated protein

**Fig. 12:**
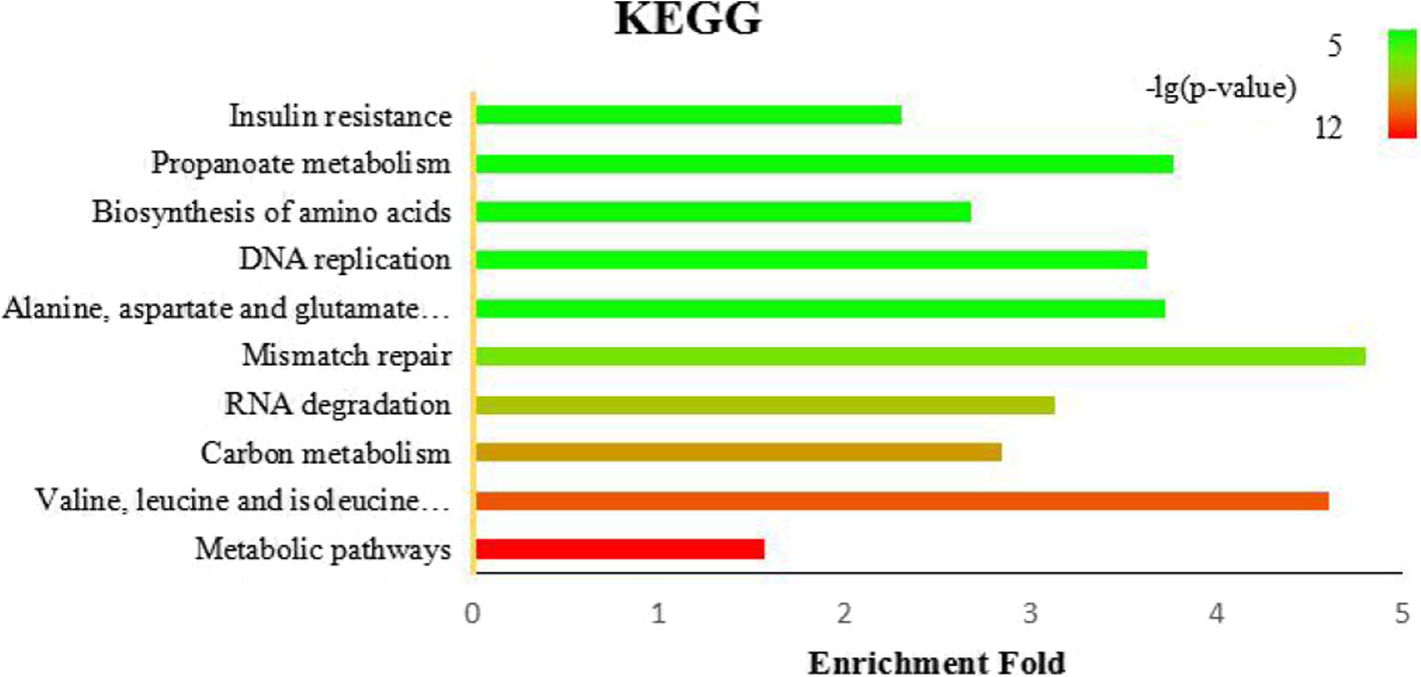
The enriched KEGG annotations for acetylated proteins (p-value< 0.01). E-fold, Enrichment fold

**Fig. 13:**
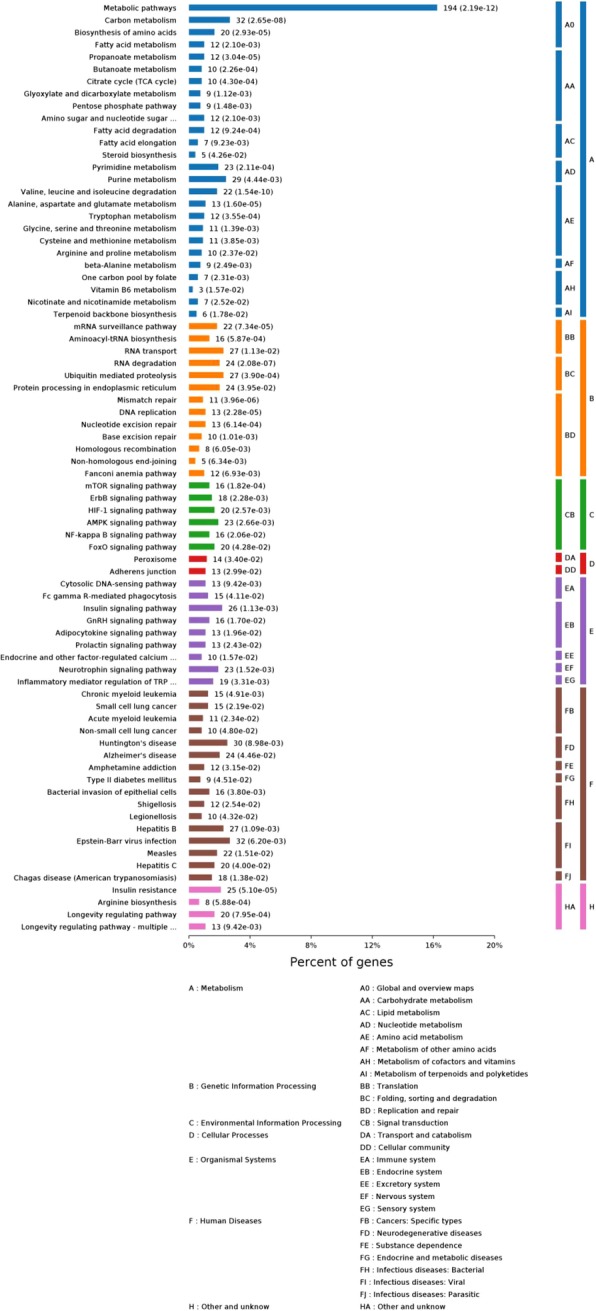
The statistical result of significant pathways(p-value< 0.01)

We map all the acetylated protein used in our study to the Kyoto Encyclopedia of Genes and Genomes (KEGG) pathways to further explore functional aspects of acetylation substrates. The top 10 enriched pathways are listed in Fig. 12 (*p*-value< 0.01) and the statistical result of significant pathways is shown in Fig. 13 (*p*-value< 0.01).

We can detect that six terms belong to metabolism class, including Metabolic pathways (hsa01100), Valine, leucine and isoleucine degradation (hsa00280), Carbon metabolism (hsa01200), Alanine, aspartate and glutamate metabolism (hsa00250), Biosynthesis of amino acids (hsa01230) and Propanoate metabolism (hsa00640), which is consistent with the result of biological process in GO, meaning that acetylation plays an important role in metabolism. And three terms are subordinate to genetic information processing class, consisting of RNA degradation (hsa03018), Mismatch repair (hsa03430) and DNA replication (hsa03030), and this observation is also clearly emerged form Fig. 13. Except metabolism pathway and genetic information processing pathway, a large portion of acetylated proteins are related to disease. Though organismal system terms and environmental information processing didn’t appear in Fig. 12, they occupy a large proportion in Fig. 13. These results concluded demonstrate that acetylation involved in diverse pathways in organism, and the study of acetylation mechanism contributes to the understanding of disease and pharmaceutical industry.

## Conclusions

In this study, we implement an application of cascade classifier to human protein acetylation prediction problem, combining sequence features and structural features. In this method, we employed a two step feature selection (mRMR and IFS). We proofed that combined feature (sequence features and structural features) is better than sequence feature, and the result of our method is much better than others’ which shown that our method is very promising and can be a useful tool to identification of acetylation sites in human. This work also indicated that cascade classifier can resolve the imbalance between positive samples and negative samples to improve the performance. We are looking forward that our method will give a powerful help for further studies of acetylation process in human body. We also test that whether different species can get good results on the same method, while the performances of other species are not satisfactory. Therefore, for future work, we are going to seek suitable methods for acetylation sites prediction in other species.

## Data Availability

All data used in our study can be downloaded from the http://plmd.biocuckoo.org/, http://cplm.biocuckoo.org/, https://www.phosphosite.org/homeAction.action, https://www.uniprot.org/, http://www.rcsb.org/.

## Abbreviations

AC: auto covariation
Acc: Accuracy
ASA: accessible surface area
GO: Gene Ontology
IE: Information Entropy
IFS: Incremental feature selection
KEGG: Kyoto Encyclopedia of Genes and Genomes
MCC: Matthew’s correlation coefficient
mRMR: maximum relevance and minimum redundancy
PCP: physicochemical property
pH: Hydrogen ion concentration
PSSM: position specific score matrix
PTMs: post-translational modifications
RC: residue composition
Sn: Sensitivity
Sp: Specificity
SS: secondary structure
SVM: Support vector machine
